# Network-Based Meta-Analyses of Associations of Multiple Gene Expression Profiles with Bone Mineral Density Variations in Women

**DOI:** 10.1371/journal.pone.0147475

**Published:** 2016-01-25

**Authors:** Hao He, Shaolong Cao, Tianhua Niu, Yu Zhou, Lan Zhang, Yong Zeng, Wei Zhu, Yu-ping Wang, Hong-wen Deng

**Affiliations:** 1 Center for Bioinformatics and Genomics, Department of Biostatistics and Bioinformatics, Tulane University School of Public Health and Tropical Medicine, New Orleans, Louisiana, United States of America; 2 Department of Biomedical Engineering, Tulane University, New Orleans, Louisiana, United States of America; University of Texas at San Antonio, UNITED STATES

## Abstract

**Background:**

Existing microarray studies of bone mineral density (BMD) have been critical for understanding the pathophysiology of osteoporosis, and have identified a number of candidate genes. However, these studies were limited by their relatively small sample sizes and were usually analyzed individually. Here, we propose a novel network-based meta-analysis approach that combines data across six microarray studies to identify functional modules from human protein-protein interaction (PPI) data, and highlight several differentially expressed genes (DEGs) and a functional module that may play an important role in BMD regulation in women.

**Methods:**

Expression profiling studies were identified by searching PubMed, Gene Expression Omnibus (GEO) and ArrayExpress. Two meta-analysis methods were applied across different gene expression profiling studies. The first, a nonparametric Fisher’s method, combined p-values from individual experiments to identify genes with large effect sizes. The second method combined effect sizes from individual datasets into a meta-effect size to gain a higher precision of effect size estimation across all datasets. Genes with *Q* test’s p-values < 0.05 or *I*^*2*^ values > 50% were assessed by a random effects model and the remainder by a fixed effects model. Using Fisher’s combined p-values, functional modules were identified through an integrated analysis of microarray data in the context of large protein–protein interaction (PPI) networks. Two previously published meta-analysis studies of genome-wide association (GWA) datasets were used to determine whether these module genes were genetically associated with BMD. Pathway enrichment analysis was performed with a hypergeometric test.

**Results:**

Six gene expression datasets were identified, which included a total of 249 (129 high BMD and 120 low BMD) female subjects. Using a network-based meta-analysis, a consensus module containing 58 genes (nodes) and 83 edges was detected. Pathway enrichment analysis of the 58 module genes revealed that these genes were enriched in several important KEGG pathways including Osteoclast differentiation, B cell receptor signaling pathway, MAPK signaling pathway, Chemokine signaling pathway and Insulin signaling pathway. The importance of module genes was replicated by demonstrating that most module genes were genetically associated with BMD in the GWAS data sets. Meta-analyses were performed at the individual gene level by combining p-values and effect sizes. Five candidate genes (*ESR1*, *MAP3K3*, *PYGM*, *RAC1* and *SYK*) were identified based on gene expression meta-analysis, and their associations with BMD were also replicated by two BMD meta-analysis studies.

**Conclusions:**

In summary, our network-based meta-analysis not only identified important differentially expressed genes but also discovered biologically meaningful functional modules for BMD determination. Our study may provide novel therapeutic targets for osteoporosis in women.

## Introduction

Osteoporosis is a skeletal disease characterized by low bone mineral density (BMD) and micro-architectural deterioration of bone which results in fragility and risk of osteoporotic fracture [1]. Hip fracture, one common and serious consequence of osteoporosis, is associated with high morbidity and mortality. It is estimated that there are 300,000 cases of hip fracture in the U.S. annually, and one in five patients will die in the year following fracture [2]. Risk of bone fracture is substantial among women with osteoporosis. Worldwide, 1 in 3 women over age 50 will experience osteoporotic fractures [3]. Most fractures occur in postmenopausal women, largely due to decreased estrogen levels, which accelerate age-related bone loss [4].

Genetic factors play an important role in the pathogenesis of osteoporosis, as evidenced by high heritability (*h*^2^) estimates of BMD ranging from 0.5–0.9 [5]. Although, many loci/genes contributing to BMD have been identified by genome-wide association (GWA) studies in recent years, those loci/genes explain only a small portion of genetic risks due to complex genetic determination. In order to search for missing heritability and to enhance our understanding of biological mechanisms, attempts have been made to identify osteoporosis risk genes as well as molecular networks that were perturbed by risk genes/loci.

Gene expression profiles across the whole genome in DNA microarrays have provided key biomarkers of osteoporosis and improved our understanding of complex gene interactions and networks during disease pathogenesis. Changes in gene profiles are associated with altered gene functions and biochemical activities. Differential gene expression analysis has revealed a number of differentially expressed genes (DEGs) in subjects with extremely discordant BMD [6–8]. The expression profiles were generated mainly from peripheral blood monocytes (PBM), B cells and bone biopsies. They have served as important models for osteoporosis research. PBM and B cells are important cell types in the immune system. Both may participate in osteoclastogenesis. The biopsies contain bone marrow cells and their precursors.

In addition, recent transcriptional co-expression network analysis has identified modules and genes that play crucial roles in the regulation of bone mass [9]. However, these studies are usually analyzed in isolation and are limited to a small number of samples. Statistically, individual gene expression profiling studies are limited by both biological (e.g., sampling of a particular population and gene expression profiles in one cell/tissue) and technical (e.g., only using one expression analysis platform) biases, leading to inconsistent results among studies and hindering the broad application of their findings and translation into clinical practice [10]. Meta-analysis approaches combining multiple gene expression datasets can increase statistical power for detecting DEGs while allowing for an assessment of heterogeneity, while also providing more robust, reproducible and accurate predictions [11]. Such meta-analysis approaches have been successfully applied to many cancers including breast cancer, lung cancer and osteosarcoma [10, 12, 13].

However, the cellular function of an individual gene can be better understood in the context of an interaction network rather than at the level of isolated components alone. Biological networks such as co-expression networks are inferred from the correlation structure of gene expression data, which is highly dependent on the threshold chosen to infer co-expression networks. Biological and technical bias may make it difficult to infer and interpret co-expression networks by combining multiple gene expression profiles [14]. However, for protein-protein interaction (PPI) networks, the edges within them represent well-defined and experimentally validated biological interactions. Recently, PPI networks have become a valuable resource for deciphering disease mechanisms based on gene expression data, as PPIs are fundamental in structuring and mediating essentially all biological processes [15]. One technique that has emerged in systems genetics to integrate multiple microarray datasets in the context of biological networks such as PPI networks is to identify functional modules (i.e., significantly differentially expressed subnetworks) within large networks [16]. These modules mark regions of the network showing striking changes in molecular that are associated with a given cellular response.

In the present study, we proposed a network-based meta-analysis framework for combining datasets from six BMD gene expression studies in women and integrating them with PPI networks. The novel part of our framework was that we not only performed meta-analysis by combining multiple gene expression datasets to increase statistical power for detecting DEGs, but also performed a computational integration of network and expression profiles to extract functional modules based on meta-analysis results. It can overcome the limited statistical power of each individual study, to resolve inconsistencies, and to lay a foundation for uncovering molecular mechanisms of osteoporosis. The first step was to identify DEGs through a meta-analysis across multiple gene expression datasets. The second was to identify functional modules from large networks by applying a nonparametric Fisher's method of combining p-values for each gene (node) in the network. Our study highlights genes that were consistently expressed differentially with statistical significance, and identifies a functional module that may play an important role in BMD regulation.

## Materials and Methods

### Dataset collection

Gene expression profiling studies were identified by searching PubMed (http://www.ncbi.nlm.nih.gov/pubmed). The following key words and their combinations were used: “osteoporosis,” “BMD,” “gene expression,” and “microarray.” The Gene Expression Omnibus (GEO) database (http://www.ncbi.nlm.nih.gov/geowebcite) and ArrayExpress (http://www.ebi.ac.uk/arrayexpress/) were also used to identify and download relevant microarray datasets through September 2014. Studies were included in the analysis if they met the following criteria: (1) case-control studies for BMD or osteoporosis in human subjects, (2) gene expression raw data available, and (3) phenotype of subjects available. We conducted this meta-analysis in accordance with the guidelines provided in the Preferred Reporting Items for Systematic Reviews and Meta-Analyses (PRISMA) statement (http://www.prisma-statement.org/) (S1 PRISMA Checklist) [17]. Raw data were downloaded from GEO and ArrayExpress websites. The following information was extracted from each identified study: Accession number, subjects, platform, number of cases and controls, tissue/cell type and raw gene expression data. Although the quantile or z-score for each study’s inclusion criteria is different, according to WHO definition [18], the low BMD group in all six studies can be classified into osteopenia/osteoporosis, while the high BMD group in all six studies can be classified into the normal.

### Data preprocessing

First, raw CEL files from each dataset were preprocessed by using the Robust Multi-array Average (RMA) algorithm to normalize and generate probe-level expression data through the *rma()* function in the R Bioconductor *affy* package [19].The RMA algorithm employs quantile normalization and smooths technical variations across samples. Second, a hierarchical clustering and principal component analysis (PCA) were performed to identify potential outliers in each dataset by using *hclust()* and *prcomp()* functions in R.

### Statistical analysis

To perform the meta-analysis, probe IDs from different platforms were annotated with their corresponding official gene symbols. When multiple probe IDs were matched to the same gene symbol, the probe ID with the largest interquartile range (IQR) of expression values among these probe IDs was selected to represent that gene symbol. This IQR-based method is preferred because it is biologically more reasonable and robust than the mean-based method, which takes the average value of expression values across multiple probe IDs [20].

Two meta-analysis methods were applied to all processed datasets. The first nonparametric meta-analysis method combined p-values from individual experiments to identify those genes with large effect sizes in all datasets. The significance analysis of microarray (SAM) method was conducted by performing 1,000 random permutations using R *samr()* package to identify DEGs between high and low BMD samples in each study [21]. Based on gene-specific *t*-statistics, this method computed a “relative difference” score for each gene, which was defined based on the ratio of change in gene expression to standard deviation in the data for that gene. SAM performed a random permutation analysis between the subjects' expression profiles to determine a null distribution. The statistical significance of each gene was computed from 1,000 permutaions. Fisher’s method was then used to combine p-values from individual experiments to identify DEGs. The combined Fisher’s statistic $\chi^{2}=-2\sum_{i=1}^{k}\text{ln}(p_{i})$ followed a *χ*^2^ distribution with 2*k* degrees of freedom (*k* is the number of datasets) under the null hypothesis (i.e., assuming null p-values are uniformly distributed). Note that smaller p-values contributed larger scores to the Fisher’s *χ*^2^ statistic.

The second method combined effect sizes across all datasets into a meta-effect size to estimate the magnitude of gene expression change. Cochran’s *Q* statistic and *I*^*2*^ were calculated as measures of between-study heterogeneity for each gene. Genes with *Q* test’s p-value < 0.05 or *I*^*2*^ > 50% were assessed by a random effects model that allows heterogeneity in the effect sizes between different datasets [22]; the remaining genes were assessed by a fixed effects model, which assumed that the standardized effect sizes can be combined across different studies and that the variations in observed effects were due only to random error [23]. Differences in each gene’s expression between high and low BMD groups were expressed as the standardized mean difference (SMD). The *z*-statistic for each gene was computed as a ratio of the pooled SMD to its standard error, and the result was compared with 1000 permutaions to obtain a nominal p-value by using R *metaDE* package [24]. P-values were corrected for multiple hypothesis testing using the Benjamini-Hochberg false discovery rate (FDR).

### Protein-protein interaction (PPI) network

A comprehensive human PPI dataset was obtained from the supplementary material of Goh et al’s study [25]. This dataset combined two high-quality systematic yeast two-hybrid experiments with PPIs obtained from published literature by manual curations [26, 27]. The PPI network constructed from this dataset included 10,174 nodes (genes) and 61,070 edges (interactions) in humans.

### Module detection

In order to identify functional modules (i.e., significantly differentially expressed subnetworks) based on an integrated analytic approach of combining multiple microarray datasets in the context of large biological PPI networks, Bioconductor *BioNet* package was applied to find an exact solution for connected subgraphs using Fisher’s combined p-values [28]. First, existing self-loops in the PPI network were removed and the largest connected component of the PPI network was selected. A binomial uniform mixture (BUM) model was fitted to the distribution of Fisher’s combined p-values, and scores were derived for these nodes at a restrictive FDR level of 0.001 [28]. Finally, these scores reflecting genes’ functional relevance were used to find the highest scoring module by using the exact Heinz (heaviest induced subgraph) method. The Heinz algorithm used the integer linear programming optimization and calculated the maximum-scoring subnetwork [16].

In order to maximally capture the variances across six microarray datasets and give a robust solution, a resampling procedure was employed to identify an optimal module containing maximally robust nodes and edges. For each iteration, we first resampled the case/control labels for each microarray dataset and calculated p-values using SAM. Fisher’s combined p-values were used in the BUM model to fit the distribution then calculate node scores at the same FDR level as used before. The highest scoring module was identified by the exact Heinz method based on the node scores. 100 iterations resulted in a total of 100 modules. These modules were used to compute consensus scores for the network and to recalculate an optimal module called “consensus module,” which contained maximally robust nodes and edges.

### Functional enrichment analysis

In order to gain further insights into the functional significance of the identified consensus module, functional enrichment analysis of KEGG pathways was performed using a hypergeometric test implemented by the WebGestalt online program (http://bioinfo.vanderbilt.edu/webgestalt/) [29]. The ‘Human Disease’ KEGG pathways category was not included in enrichment analysis because of lack of its direct relevance to the present study.

### Important candidate gene selection and validation

In order to prioritize candidate genes from the consensus module, and to decipher their biologically meaningful functions, meta-analyses were applied by combining effect sizes and combining p-values at the individual gene level. Important candidate genes were selected based on the following criteria: (1) p-value < 0.05 for meta-analysis of effect sizes, (2) p-value < 0.05 for Fisher’s method of combining p-values, and (3) presence in the consensus module. To determine whether these selected candidate genes were genetically associated with BMD in larger human populations, we used data from the two largest previously published meta-analyses of GWA datasets. The first was the meta-analysis from the Genetic Factors for Osteoporosis (GEFOS) Consortium (GEFOS-2). It is the largest meta-analysis to date in the field of bone density, including 17 GWASs and 32,961 individuals of European and East Asian ancestry [30]. The second was from an imputation-based meta-analysis (Meta7), including seven GWA studies consisting of 11,140 subjects for BMDs at lumbar spine, hip, and femoral neck. Details of the statistical analysis were provided previously [31]. The list of single-nucleotide polymorphisms (SNPs) and their meta-analysis p-values for various BMD traits in women were obtained. The most significant SNP for a given gene was chosen as a gene level p-value. Associated genes were defined as those with nominal p-values ≤  0.05 for at least one of the BMD traits.

## Results

### Studies included in the meta-analysis and data preprocessing

**Fig 1:**
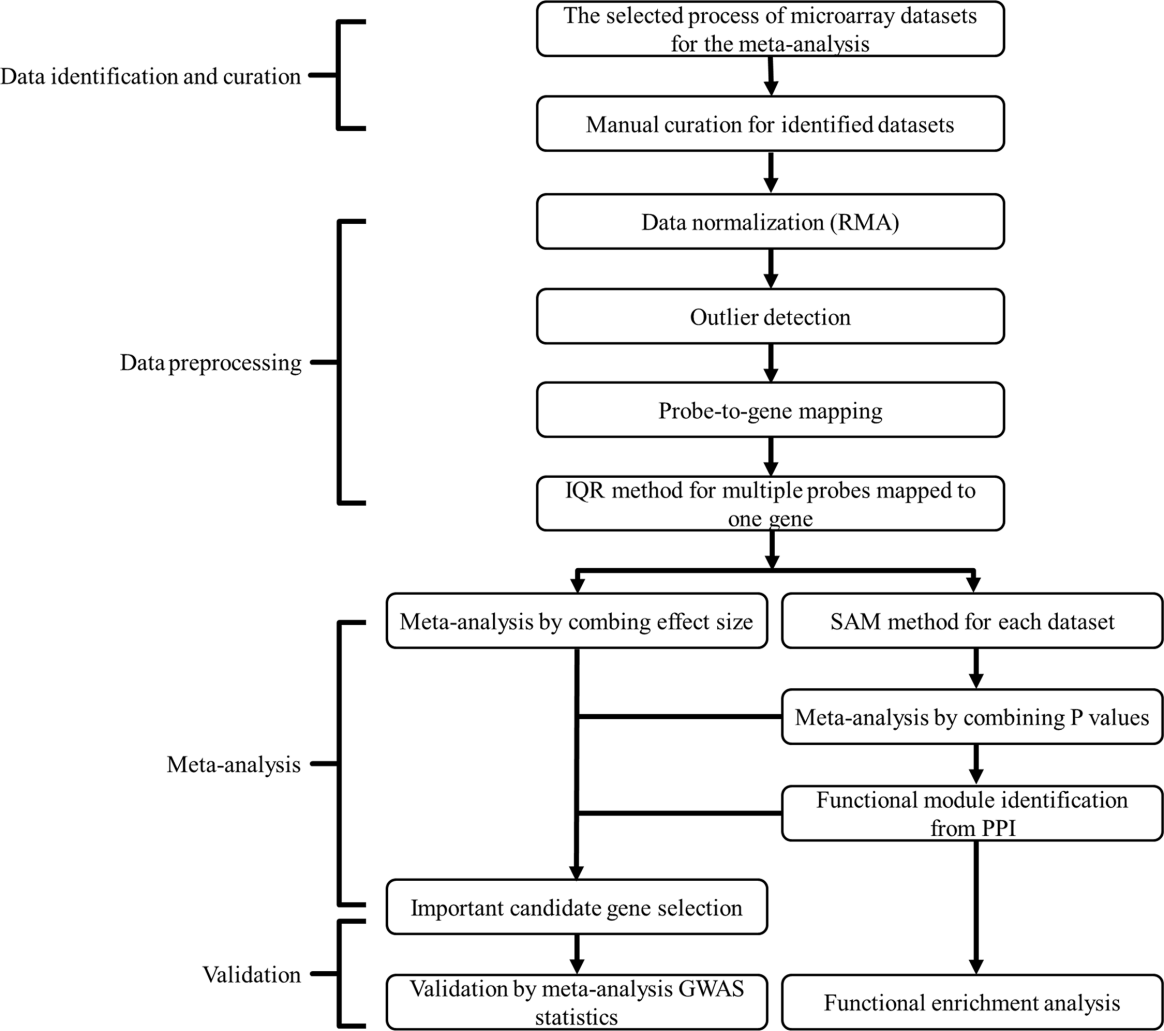
The workflow of network-based meta-analysis.

We proposed a network-based meta-analysis framework as outlined in Fig 1. The six gene expression profiling studies were carefully identified and downloaded from GEO and ArrayExpress. Three datasets consisted of expression profiles generated from peripheral blood monocytes (PBMs), and two generated from B cells, which were isolated and purified in subjects with low versus high hip BMD values. Subjects of these five gene expression profiling studies were all recruited for the same purpose of systemically searching for DEGs underlying BMD variations. The sixth study was based on 84 bone biopsies from postmenopausal women, and was aimed at identifying important genes associated with BMD variations. Of these, 45 had high hip BMD and 39 had low hip BMD. Two gene expression platforms were used for expression profiling, the Affymetrix U-133 Plus 2.0 Gene Chips and the Affymetrix U-133A Gene Chips (Affymetrix, Santa Clara, CA, USA). Overall, these six datasets included 249 (129 high BMD and 120 low BMD) female subjects. The datasets were normalized individually using RMA algorithm. The outliers in each dataset were then detected and then removed. Annotation files for both microarray platforms were downloaded from the Affymetrix website and used to map probe IDs to unique gene symbols in each dataset. When multiple probe IDs were matched to an identical gene symbol in a dataset, the probe ID with the largest IQR of expression values among all multiple probe IDs was selected to represent the corresponding gene symbol. After these data preprocessing steps, we were left with one gene per probe ID per dataset. This led to a total of 13,341 common genes shared by both gene expression platforms. Each gene was then subjected to meta-analysis across the datasets. The detailed characteristics of study samples, type of gene chip and number of outliers are shown in Table 1.

**Table 1:** Characteristics of the individual studies.

Accession number	Subject	Race	Sample size(High: LowBMD)	Outlier removed(High: LowBMD)	Platform	Tissue/cell	Samplesource
GSE56815	Pre- and postmenopausal female	Caucasian	80(40:40)	3(2:1)	GPL96 HG-U133A	Peripheral blood monocytes	in vivo
GSE7158	Premenopausal female	Chinese	26(14:12)	2(2:0)	GPL570 HG-U133_Plus_2	Peripheral blood monocytes	in vivo
GSE2208	Pre- and postmenopausal female	Caucasian	19(10:9)	0(0:0)	GPL96 HG-U133A	Peripheral blood monocytes	in vivo
E-MEXP-1618	Postmenopausal female	Caucasian	84(45:39)	4(1:3)	GPL570 HG-U133_Plus_2	Bone biopsies	in vivo
GSE7429	Postmenopausal female	Caucasian	20(10:10)	3(2:1)	GPL96 HG-U133A	Circulating B cells	in vivo
GSE13850	Postmenopausal female	Caucasian	20(10:10)	1(0:1)	GPL96 HG-U133A	Circulating B cells	in vivo

Note: HG-U133_Plus_2: Affymetrix Human Genome U133 Plus 2.0 Array

HG-U133A: Affymetrix Human Genome U133A Array

### Network-based meta-analysis

Two meta-analysis approaches were applied to analyze these six preprocessed microarray datasets. In brief, the first meta-analysis approach combined p-values across individual datasets using Fisher’s method to identify DEGs in all datasets. P-values from individual microarray datasets were derived from the SAM method in R *samr* package with 1,000 random permutations. 2,244 out of 13,341 genes were identified by a FDR-adjusted p-value (q-value) of 0.05, and 508 by a more stringent cutoff q-value 0.01 (S1 Table).

The second meta-analysis approach combined effect sizes from six studies implemented in the *metaDE* package. It led to the identification of genes that were upregulated and downregulated, and 22 significant DEGs were those displaying q-value < 0.05 (S2 Table). The most significant gene was *PYGM* (nominal p-value = 2.25×10^−7^, q-value = 0.003).

**Fig 2:**
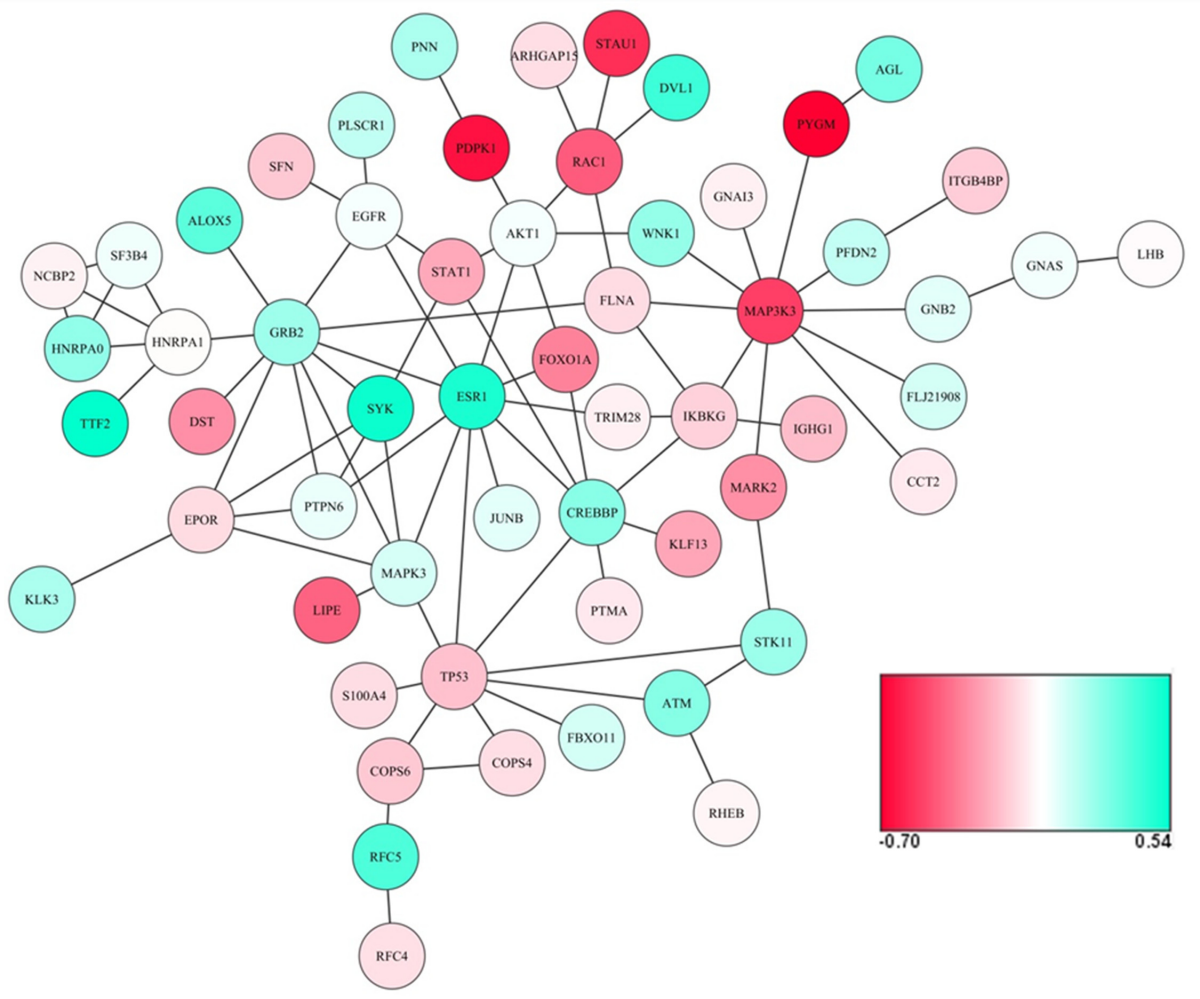
Consensus module. Differential expressed genes between high and low BMD are shown in red and green, where green color is indicative of a positive pooled SMD, an upregulation in high BMD, and red color is indicative of a negative pooled SMD, an upregulation in low BMD.

The Bioconductor package *BioNet* was used for an exact solution to find connected subgraphs from the large PPI network, using Fisher’s combined p-values at a restrictive FDR of 0.001. After 100 iterations, a consensus module was detected containing 58 genes (nodes) and 83 edges, shown in Fig 2. The consensus module captured the characteristically differentially-expressed interaction modules associated with BMD variations in women.

**Fig 3:**
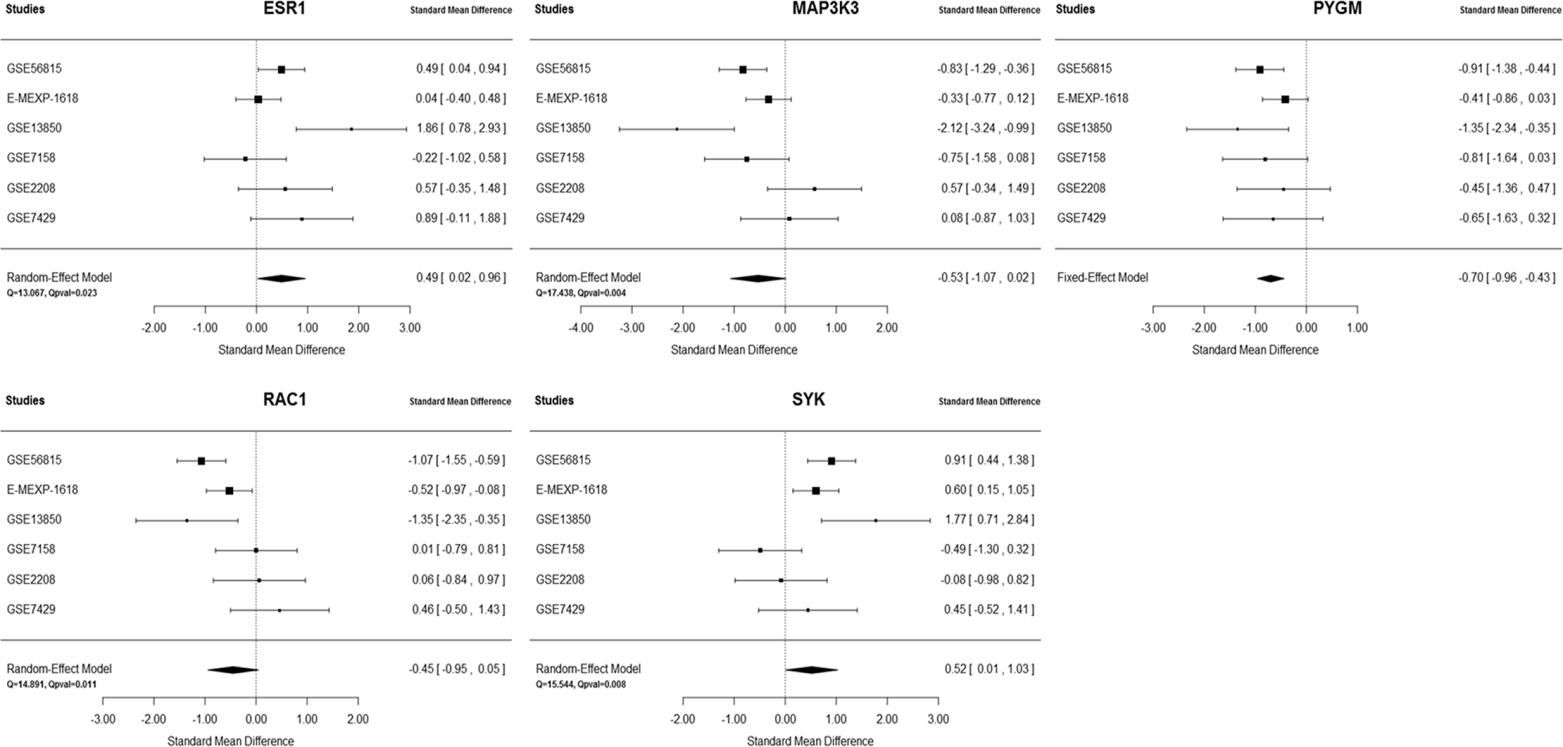
Forest plots for five candidate genes across six gene expression profiling datasets.

Through the candidate gene selection criteria, five genes, *ESR1*, *MAP3K3*, *PYGM*, *RAC1*and *SYK*, were identified. The forest plots for each gene across six datasets are shown in Fig 3. As these genes may play important roles in controlling module behavior and thus in BMD regulation, two published meta-analysis GWAS were used to test whether these genes were genetically associated with BMD. Table 2 shows the results for two meta-analysis approaches and gene-level p-values in both GEFOS2 and Meta7, represented by the most significant SNP/marker in the gene. Genes in the consensus module with nominal p-value ≤ 0.05 are shown in S3 Table.

**Table 2:** Results for five candidate genes.

Genes	SMD (SD)	Z-statistic	P-value	Fisher’s statistic	P-value	Gene level p-value in GEFOS2	Gene level p-value in Meta7
*ESR1*	0.49 (0.24)	2.05	2.67E-02	31.81	1.48E-03	1.13E-11	2.52E-07
*MAP3K3*	-0.53 (0.28)	-1.90	3.95E-02	46.93	4.79E-06	2.25E-02	2.65E-02
*PYGM*	-0.70 (0.13)	-5.17	2.25E-07	42.85	2.40E-05	2.67E-03	6.67E-04
*RAC1*	-0.45 (0.25)	-1.76	5.50E-02	40.90	5.10E-05	2.74E-02	4.41E-02
*SYK*	0.52 (0.26)	1.99	3.13E-02	49.72	1.57E-06	2.51E-03	3.84E-02

Note: SMD, standardized mean difference; SD, standard deviation.

### Functional enrichment analysis

Table 3 summarizes the results of enrichment analysis of 58 genes in the consensus module. Even after Bonferroni correction, a number of KEGG pathways remained significantly enriched. The most significant pathway was osteoclast differentiation (p-value = 3.82E-12). Other top enriched pathways included the B cell receptor signaling pathway, MAPK signaling pathway, Chemokine signaling pathway and Insulin signaling pathway.

**Table 3:** KEGG pathway enrichment results for genes in the consensus module.

KEGG	Genes	p-value^a^	Adjusted p-value^b^
Osteoclast differentiation	*GRB2*, *IKBKG*, *AKT1*, *JUNB*, *RAC1*, *STAT1*, *SYK*, *MAPK3*	3.82E-12	3.44E-11
B cell receptor signaling pathway	*GRB2*, *IKBKG*, *AKT1*, *PTPN6*, *SYK*, *RAC1*, *MAPK3*	5.22E-12	3.76E-11
MAPK signaling pathway	*GRB2*, *MAP3K3*, *IKBKG*, *AKT1*, *RAC1*, *TP53*, *FLNA*, *EGFR*, *MAPK3*	4.22E-11	2.53E-10
Chemokine signaling pathway	*GRB2*, *IKBKG*, *AKT1*, *RAC1*, *STAT1*, *GNAI3*, *GNB2*, *MAPK3*	8.78E-11	3.51E-10
Insulin signaling pathway	*GRB2*, *PDPK1*, *LIPE*, *AKT1*, *PYGM*, *RHEB*, *MAPK3*	4.02E-10	1.32E-09
GnRH signaling pathway	*GNAS*, *GRB2*, *MAP3K3*, *LHB*, *EGFR*, *MAPK3*	2.98E-09	8.94E-09
mTOR signaling pathway	*PDPK1*, *STK11*, *AKT1*, *RHEB*, *MAPK3*	5.74E-09	1.48E-08
Neurotrophin signaling pathway	*GRB2*, *MAP3K3*, *AKT1*, *RAC1*, *MAPK3*, *TP53*	1.19E-08	2.86E-08
Adherens junction	*CREBBP*, *PTPN6*, *EGFR*, *RAC1*, *MAPK3*	3.26E-08	5.87E-08
Jak-STAT signaling pathway	*GRB2*, *CREBBP*, *EPOR*, *AKT1*, *PTPN6*, *STAT1*	3.90E-08	6.69E-08

^a^ hypergeometric test p-value

^b^ Bonferroni correction adjusted p-value

To further understand those five candidate gene functions, we first characterized the broad tissue-specific pattern of mRNA expression profiles using microarray data from 79 human tissues and cell types (GeneAtlas U133A, at http://biogps.org/) [32]. Only *PYGM* and *MAP3K3* were found to have tissue-specific pattern and were highly expressed in skeletal muscle and monocyte, respectively. Then we checked the tissue-specific patterns in a microarray data from 96 mouse tissues and cell types (GeneAtlas MOE430), which contains more bone-related cells, such as B-cells, T-cells, bone marrow, osteoclast, osteoblast and bone[33]. Specifically, *ESR1* and *PYGM* genes were highly expressed in uterus and skeletal muscle, respectively. *MAP3K3* and *RAC1* genes were highly expressed in bone marrow and osteoclasts, respectively. *SYK* was expressed in multiple tissues and cell types, including B cells, marcrophages and bone marrow (S1 Fig).

## Discussion

A comprehensive analysis of the mechanism underlying osteoporosis development is crucial for developing rational treatment options. Many transcriptional studies for osteoporosis have been conducted; however, most of them had limited sample sizes, making it challenging to characterize the molecular and cellular events during the pathogenesis of osteoporosis. In addition, experimental confounders such as platform variability and cell/tissue-specific profiles were problematic in individual experiments. In this paper, we have contributed the largest meta-analysis to date of gene expression in osteoporosis. We proposed a network-based meta-analysis approach that combines meta-analyses across six microarray datasets, and functional module identification from human PPI. The study highlights genes that were consistently expressed differentially with statistical significance, and identifies a functional module that may play an important role in BMD regulation.

Six gene expression datasets were identified and raw CEL files were downloaded from GEO and ArrayExpress. All were based on the Affymetrix U-133 series platform; specifically, four on Affymetrix U-133A Gene Chips and two on Affymetrix U-133 Plus 2.0 Gene Chips. 13,341 unique genes were shared by both platforms. In total there were 249 (129 high BMD and 120 low BMD) female subjects. Subjects in five datasets were all recruited for the same purpose of systemically searching for DEGs underlying BMD variations. Three datasets were generated from PBM and two from B cells. The sixth was generated from bone biopsies. As important cell types in the immune system, PBM and B cells may both participate in osteoclastogenesis and have served as important models for osteoporosis research [6, 7, 34]. First, PBMs are osteoclast progenitor cells and produce a wide variety of factors such as interleukin 1 (IL-1), IL-6, tumor necrosis factor (TNF) and transforming growth factor beta (TGF-β) for osteoclastogenesis and bone resorption [35–37]. Second, B cell precursors can differentiate into osteoclasts in vitro, and estrogen deficiency may enhance osteoclastogenesis by increasing the number of B cell precursors with the potential for osteoclastic differentiation [38–40]. Osteoprotegerin (OPG), as a decoy receptor competing with RANK, can bind to the key osteoclastogenic cytokine RANKL and block its effect on osteoclastogenesis [41]. Human B cells secrete OPG, and under certain physiological conditions B lineage cells are the dominant source of OPG in mouse bone marrow [40, 42]. The gene expression study (E-MEXP-1618) performed by Reppe and colleagues is the most extensive transcriptome analysis of bone biopsies to date [8]. The biopsies contain bone marrow cells belonging to hematological, immunological, endothelial and stromal cell lineages in addition to bone cells and their precursors [8].

Experimental confounders such as platform variability and tissue-specific profiles could overwhelm expression measurements in individual experiments. It was hypothesized that irrespective of datasets analyzed, a set of genes that were significantly differentially expressed would constitute a robust gene expression signature of disease across multiple independent studies [10]. Therefore, by integrating six microarray datasets from different tissues/cell types in 249 female subjects, we were able to increase sample size, avoid tissue/cell type-specific bias through a novel network-based meta-analysis, and identify significantly expressed genes with functional relevance to BMD, as well as a consensus functional module, which elucidated specific molecular processes underlying BMD variation.

Gene expression profiles are useful but insufficient for identifying DEGs. Proteins, the main agents of biological function that usually operate in complexes and PPIs, are critical for healthy and diseased states in organisms, which in turn can form a molecular basis for diagnosis, prevention and treatment [43]. More and more molecular studies have benefited from combining gene expression profiles and PPI data, which allows the detection of previously unknown dysregulated modules within the global PPI network [16]. These modules contain many DEGs that may be missed by gene expression profile analysis based on restrictive significance threshold. Further, they could provide researchers with an in-depth understanding of the regulatory processes underlying the observed changes in gene expression [44]. Our network-based meta-analysis approach used summary statistics across six gene expression profiles to identify functional modules in a large human PPI [45]. A consensus module containing 58 genes was revealed across six datasets to contribute to BMD variations. This consensus module captured the characteristically differentially-expressed interaction modules associated with BMD variations in women. KEGG pathway enrichment analysis was performed for these 58 genes to seek evidence of their potential involvement in BMD regulation. The top enriched pathways included Osteoclast differentiation, B cell receptor signaling pathway, MAPK signaling pathway, Chemokine signaling pathway and Insulin signaling pathway. These top KEGG pathways, especially Osteoclast differentiation, linked the consensus module to regulation of BMD. Eight genes in the consensus module (*GRB2*, *IKBKG*, *AKT1*, *JUNB*, *RAC1*, *STAT1*, *SYK* and *MAPK3*) were contained in the Osteoclast differentiation pathway. Interestingly, one candidate gene, *RAC1*, was highly expressed in mouse osteoclasts.

With respect to these 58 module genes, meta-analyses were applied at the individual gene level by combining effect sizes and p-values to prioritize candidate genes and provide biologically meaningful interpretations. According to the criteria, five candidate genes (*ESR1*, *MAP3K3*, *PYGM*, *RAC1* and *SYK*) were identified, and their associations with BMD were replicated by both BMD meta-analysis studies. *ESR1* is a well-known candidate gene for osteoporosis. It is the major receptor mediating estrogen action in bone tissue, and it has a prominent effect on the regulation of bone turnover and the maintenance of bone mass [46]. The pooled SMD across six gene expression profiling datasets showed that *ESR1* was upregulated in the high BMD group but not the low BMD group, which was consistent with previous findings [34]. Both *SYK* and *RAC1* were contained in the Osteoclast differentiation pathway. As an important protein tyrosine kinase, *SYK* plays an indispensable role for osteoclast function by regulating α-tubulin deacetylation [47]. A previous study demonstrated that *RAC1* is critically involved in osteoclast differentiation through TNF-related activation-induced cytokine (TRANCE)-induced nuclear factor (NF)-kappaB activation [48]. *MAP3K3* was also found to play a critical role in TNF-induced NF-kappaB activation [49]. The interaction of *MAP3K3* with *PYGM* (shown in Fig 2) was also reported by another study on the human TNF-alpha/NF-kappaB signal transduction pathway [50]. As all five candidate genes were contained in the consensus module and interacted with other module genes, their functional relevance in BMD regulation not only supports the robustness of our current study, but also provides important new information for understanding the pathogenesis of osteoporosis and identifying potentially novel therapeutic targets.

The present study has some limitations. First, although all subjects in six datasets were female, some confounding factors like age, height, weight and menopausal status were not controlled in the meta-analysis. Second, only common genes shared by both gene expression platforms were included into the meta-analysis. About 7000 genes which only exist in HG-U133_Plus_2 array were discarded, which may result in missing novel findings in our study. Third, our study lacked subsequent cellular and molecular experiments to validate the biological functions of the five candidate genes and the consensus module. Despite these limitations, our findings still have important implications for the molecular mechanisms of osteoporosis, and further experimental research is still needed to confirm our study.

In summary, our network-based meta-analysis not only identified important DEGs by increasing sample size and accounting for biases inherent in single gene expression studies, but also discovered functional modules biologically related to osteoporosis pathology. Our study may provide important potential therapeutic targets for osteoporosis. With the increasing availability of public gene expression data, our approach could have broader applications to many complex diseases and may accelerate translational research.

## Supporting Information

S1 PRISMA Checklist (DOC)

S1 Fig Tissue-specific expression pattern of five candidate genes in microarray data from 96 mouse tissues and cell types. (PDF)

S1 Table Results of meta-analyisis using Fisher's methods. (XLSX)

S2 Table Results of meta-analyisis using effect size combined methods. (XLSX)

S3 Table Significant genes in the consensus module associated with BMD in GEFOS2 and Meta7. (XLSX)
