# Supplementary figures and images for: Network-Based Meta-Analyses of Associations of Multiple Gene Expression Profiles with Bone Mineral Density Variations in Women
Source: PLoS One. 2016 Jan 25;11(1):e0147475. doi: 10.1371/journal.pone.0147475 (PMC4726665; doi:10.1371/journal.pone.0147475)

ESR1

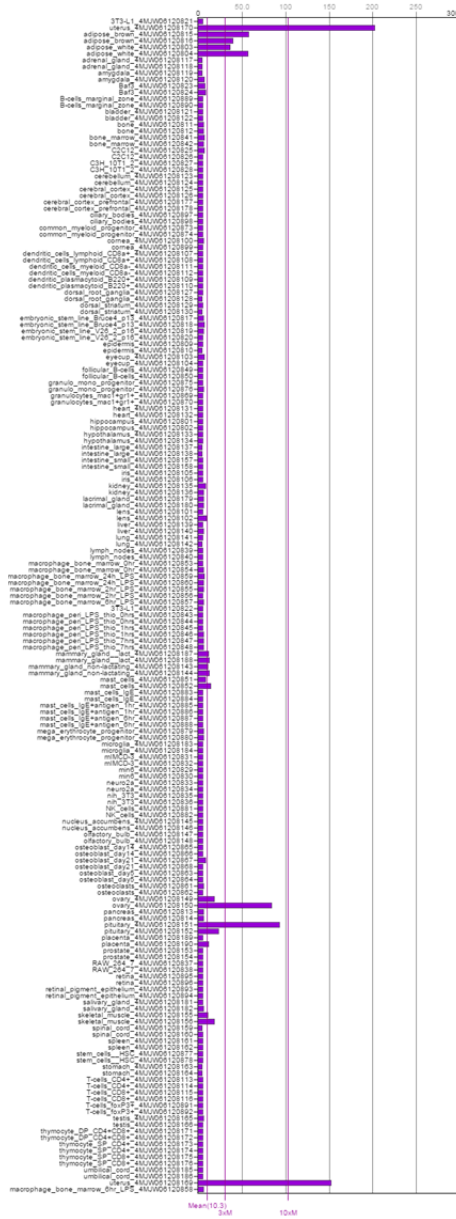

MAP3K3

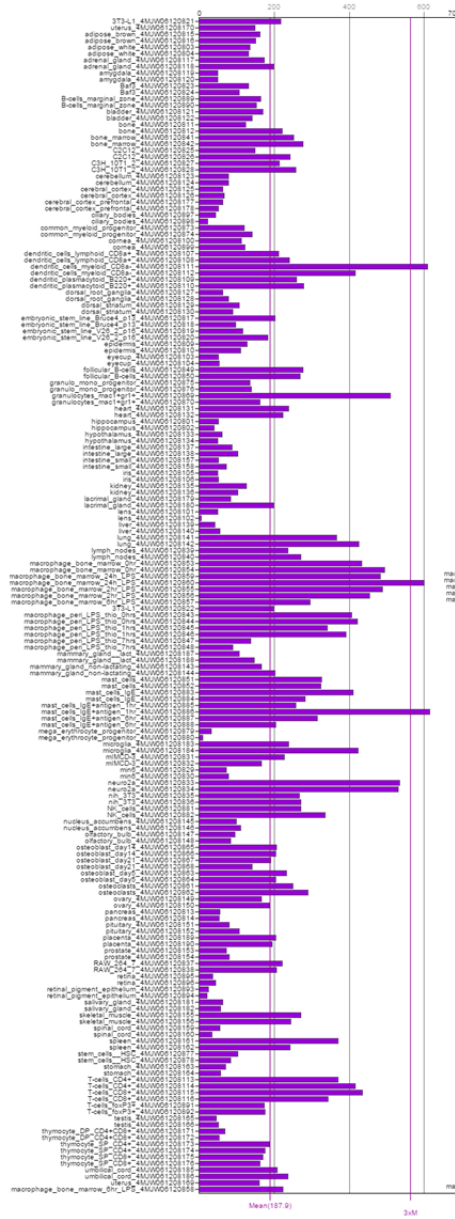

PYGM

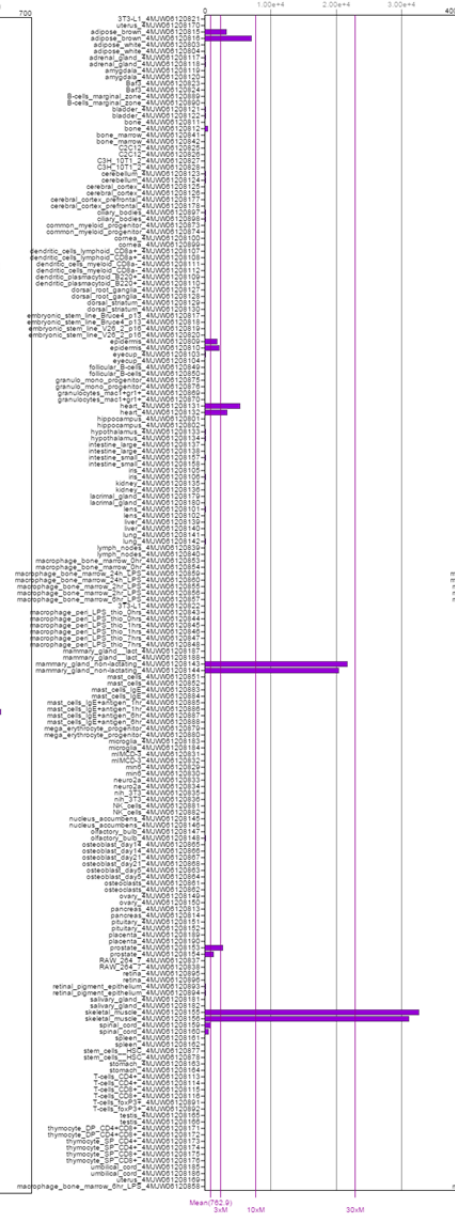

RAC1

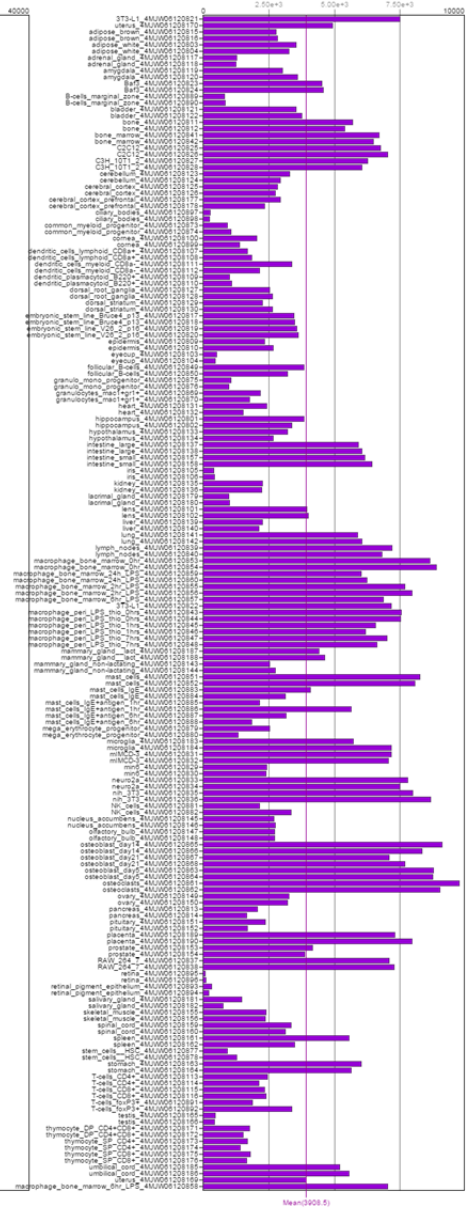

*SYK*

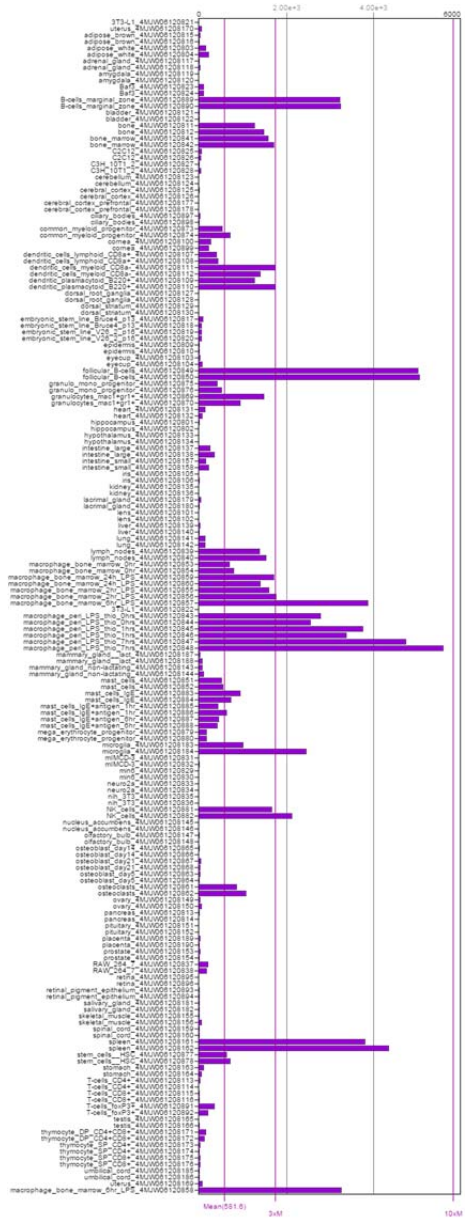

Supplement: S1 Fig — (PDF) [file pone.0147475.s002.pdf]
